# Prof. Laugier

**Published:** 1864-09

**Authors:** 


PROF. LAUGIER, one of the surgeons of the Hotel Dieu, has recently made a most important communication to the Academy of Sciences. In an operation performed on the arm, and in which the median nerve had been severed, that skilful surgeon united by a suture the two ends of the nerve. Almost immediately after signs of sensibility were observed, and in a few days more the nerve had entirely recovered all its properties of sensation and motion. I need not insist on the importance of this case, which throws such a new light on physiological pathology of the nervous system. Within a few weeks, in a discussion which took place at the Society of Surgery, it was affirmed by several members that the regeneration of the nervous tubes, which alone could cause the recovery of sensibility and motility, was the work of weeks and months, and could not immediately take place. Such, also, was the opinion of M. Brown-Sequard and of M. Vulpian and Philippeaux. These two gentlemen published last year a memoir which received academical honors, and in which they gave the relation of different experiments they had made, the result of which is entirely opposed to that obtained by M. Laugier.—*Paris Cor. of Lond. Lancet.*

